# Genetic visualization of notch signaling in mammalian neurogenesis

**DOI:** 10.1007/s00018-012-1151-x

**Published:** 2012-09-13

**Authors:** Itaru Imayoshi, Hiromi Shimojo, Masayuki Sakamoto, Toshiyuki Ohtsuka, Ryoichiro Kageyama

**Affiliations:** 1 Institute for Virus Research, Kyoto University, Shogoin-Kawahara, Sakyo-ku, Kyoto, 606-8507 Japan; 2 The Hakubi Center, Kyoto University, Kyoto, Japan; 3 Japan Science and Technology Agency, Precursory Research for Embryonic Science and Technology (PRESTO), Kyoto, 606-8507 Japan; 4 Japan Science and Technology Agency, Core Research for Evolutional Science and Technology (CREST), Kyoto, 606-8507 Japan

**Keywords:** Notch, NICD, Hes, Hey, Neurogenesis, Adult neurogenesis, Fluorescent reporter, Luciferase reporter

## Abstract

Notch signaling plays crucial roles in fate determination and the differentiation of neural stem cells in embryonic and adult brains. It is now clear that the notch pathway is under more complex and dynamic regulation than previously thought. To understand the functional details of notch signaling more precisely, it is important to reveal when, where, and how notch signaling is dynamically communicated between cells, for which the visualization of notch signaling is essential. In this review, we introduce recent technical advances in the visualization of notch signaling during neural development and in the adult brain, and we discuss the physiological significance of dynamic regulation of notch signaling.

## Introduction

**Fig. 1:**
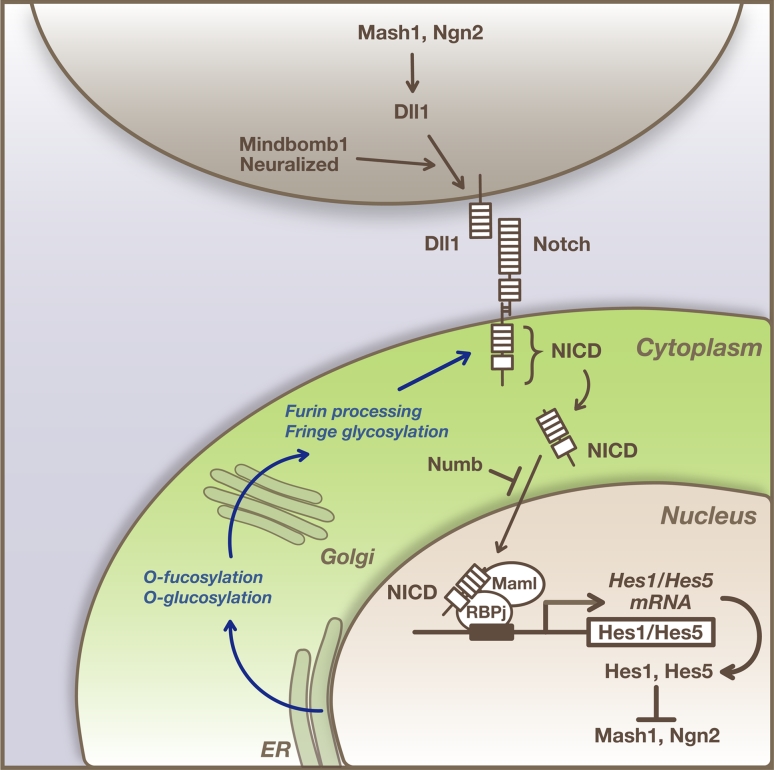
Notch signaling pathway. The proneural genes *Mash1* and *Ngn2* induce expression of notch ligands such as Dll1, which activate notch signaling in neighboring cells. Upon activation, the notch intracellular domain (NICD) is released from the transmembrane region and transferred into the nucleus, where NICD forms a complex with RBPj and induces *Hes1* and *Hes5* expression. Hes1 and Hes5 repress proneural gene expression. During maturation and trafficking to the cell surface, notch receptors undergo furin processing and glycosylation, which can impact their responsiveness to their ligands. The ligand-induced activation of notch signaling is dynamically regulated by endocytic trafficking, which can be modulated by the different ubiquitin ligases, such as Mind bom and Neuralized

The notch signaling pathway is well known as an important signaling mechanism for communication between neighboring cells [1–3]. Many of the components in the notch signaling pathway have been identified, and the pathway is important for neural stem cell (NSC) maintenance and differentiation [4, 5]. The notch pathway plays pivotal roles in a process referred to as “lateral inhibition”, which ensures that cells differentiate into distinct cell types from an initially homogenous cell population (Fig. 1) [2, 6]. In the germinal zone of developing mammalian brains, NSCs initially undergo proliferation only, and then subsets of cells start neuronal differentiation, while others remain as NSCs. During this process, proneural genes, such as *Mash1/Ascl1* and *Neurogenins* (*Ngn1*, *Ngn2*), are expressed by a subset of NSCs thereby inducing the neuronal differentiation. In those cells undergoing neuronal differentiation, notch ligands such as Delta-like1 (Dll1) are up-regulated, which in turn activate notch signaling in neighboring cells. As a result of notch activation, neuronal differentiation is inhibited in neighboring cells and they remain as NSCs [7, 8].

The roles of notch signaling in NSCs were studied mainly during embryonic development, but there is growing evidence that it also plays essential roles in the maintenance and differentiation of adult NSCs [4, 9, 10]. Their ability to continuously generate new neurons over time depends on the coordinated balance of stem cell maintenance and differentiation. Incomplete maintenance and premature neuronal differentiation will deplete the NSC pool and, consequently, reduce the supply of new neurons. On the other hand, increased stem cell maintenance at the expense of proper neuronal differentiation will also impair the ability of NSCs to generate a sufficient number of new neurons. Accumulating evidence indicates that a notch-dependent pathway underlies the central molecular mechanism regulating this tight balance between NSC maintenance and differentiation in the adult brain [11–15].

Recent studies revealed that notch signaling is under the control of more complex and dynamic regulation than previously thought. In this review, we introduce the recent technical progress made in visualizing notch signaling, and discuss recent advances in understanding when, where, and how notch signaling is regulated during neural development and in the adult brain.

## Neurogenesis in the developing and adult forebrain

NSCs of the lateral ventricular wall of the forebrain undergo changes in morphology and produce different progeny as the brain development proceeds [16]. NSCs begin as neuroepithelial cells, become radial glial cells, and then finally have many astrocytic characteristics in the adult brain [17].

**Fig. 2:**
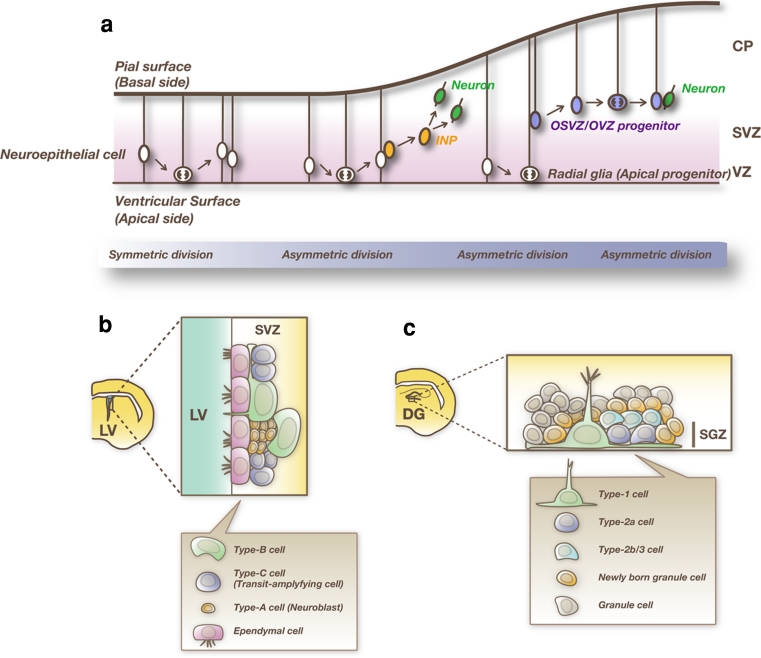
Neurogenesis in the developing and adult forebrain. **a** Differentiation of NSCs in the embryonic dorsal telencephalon. NSCs initially undergo symmetric cell division and proliferate extensively. Then, these cells give rise to neurons or intermediate neural progenitors (INPs) by asymmetric cell division. Neurons and INPs migrate into the cortical plate (CP) and the subventricular zone (SVZ), respectively. INPs further divide in the SVZ and produce more neurons. Some NSCs become outer SVZ (OSVZ) or outer VZ (OVZ) progenitors, which have radial fibers that extend to the pial surface but lack apical processes. After production of neurons, NSCs finally differentiate into glial cells. **b** Neurogenic niche of the subventricular zone (SVZ) of the lateral ventricle (LV) of the adult brain. In this region, NSCs (type B cells) exist and new neurons are continuously generated. Type B cells are GFAP-positive cells with the structural and molecular characteristics of astrocytes. Type B cells divide to generate transit-amplifying cells (type C cells), which in turn differentiate into neuroblasts (type A cells) that migrate into the olfactory bulb. **c** Adult neurogenesis in the dentate gyrus (DG) of the hippocampus. Type 1 and type 2a progenitor cells in the subgranular zone (SGZ) are shown. These progenitor cells give rise to transit-amplifying cells (type 2b/type 3 cells). Then, transit-amplifying cells differentiate into dentate granule cells through several maturation steps

At an early developmental stage, neuroepithelial cells initially undergo symmetric cell division in the apical-most region, the ventricular zone (VZ) of the embryonic forebrain (Fig. 2a). Neuroepithelial cells are transformed into radial glial cells at the onset of neurogenesis [18–22]. During the peak phase of neurogenesis, around embryonic day 13–18 (E13–E18) in mice, radial glial cells undergo asymmetric cell division; each radial glial cell divides into two distinct cell types, one radial glial cell and one immature neuron or an intermediate neural progenitor (INP). Immature neurons migrate outside of the VZ into the outer layers, where they become mature neurons, while INPs migrate into the subventricular zone (SVZ), proliferate further, and give rise to more neurons. Some cells in the SVZ, called outer SVZ (OSVZ) or outer VZ (OVZ) progenitors, have radial processes that extend to the pial surface but lack apical end feet [23–26]. Like radial glial cells, OSVZ/OVZ progenitor cells predominantly undergo asymmetric division to self-renew while simultaneously giving rise either to a immature neuron or to an INPs. After producing neurons during development, NSCs finally differentiate into astrocytes, oligodendrocytes, and ependymal cells. Some of NSCs are maintained in the adult brain, where they exist principally in two regions: the SVZ of the lateral ventricle and the subgranular zone (SGZ) of the hippocampal dentate gyrus, where neurogenesis occurs continuously [27, 28].

The SVZ is a layer extending along the lateral wall of the lateral ventricle, where active cell proliferation continuously occurs (Fig. 2b) [29]. Neurons formed in the SVZ migrate via the rostral migratory stream into the olfactory bulb and become local inhibitory interneurons (granule cells and periglomerular cells) [30]. A subset of cells that have the astrocyte-like features and express glial fibrillary acidic protein (GFAP), a canonical astrocyte marker protein, (type B cells) function as NSCs in the adult SVZ [31]. Type B cells divide slowly and give rise to rapidly proliferating “transit-amplifying cells” (type C cells), which then generate migrating neuroblasts (type A cells) after several cell divisions.

In the SGZ of the adult hippocampal dentate gyrus, type 1 NSCs have astrocytic features and are marked by GFAP (Fig. 2c) [32]. Although these cells have proliferative capacity, they cycle much slower than the type 2a progenitor cells that follow. While Nestin, Sox2, and brain lipid-binding protein (BLBP) are also expressed in type 1 cells, the expression persists in type 2a cells. NeuroD and Doublecortin (Dcx) appear in type 2b, the later stage of type 2 cells, and persist in postmitotic but immature granule cell precursors. Neurons born in the SGZ migrate into the granule cell layer and become granule cells of the dentate gyrus [33]. Thus, NSCs give rise to neurons and glial cells during development and in the adult brain [17].

## The molecular nature of the notch signaling pathway

Notch signaling is regulated by cell–cell interactions that occur via notch receptors, of which there are four in mammals (NOTCH1–4), and their ligands, the Delta-like (Dll1, 3, and 4) and Jagged (Jag1 and Jag2) proteins (Fig. 1) [1]. Newly translated notch receptor proteins are glycosylated by the GDP-fucose protein *O*-fucosyl-transferase 1 (POFUT1) and three β1,3-GlcNAc-transferases (lunatic fringe, manic fringe, and radical fringe), which are essential for the production of fully functional receptors and can influence their responsiveness to their ligands [34]. During maturation and trafficking to the cell surface membrane, notch receptors undergo proteolytic cleavage by furin at site 1 (S1), which converts the notch polypeptide into a heterodimer, composed of the notch extracellular domain and the notch transmembrane/intracellular domain connected by noncovalent interactions [35].

A wide range of notch ligands bind to and activate the notch receptor. The activity and availability of notch receptors and ligands are also regulated by endocytic trafficking, which can be modulated by various ubiquitin ligases [36]. Upon activation by ligands such as Dll1 on neighboring cells, notch receptors are cleaved by ADAM-family metalloproteases at site 2 (S2). The truncated transmembrane/intracellular domains are subjected to the further proteolytic events by presenilin proteases, such as PSEN1 or PSEN2, of the γ-secretase complex. γ-secretase cleaves the notch transmembrane domain progressively from site 3 (S3) to site 4 (S4).

The cleaved intracellular domain of the notch receptor (NICD) is released from the cell membrane and translocated to the nucleus, where it associates with the DNA binding protein RBPj/CBF-1 and other transcriptional coactivators, such as Maml1–3, to bind and activate downstream target genes [1]. In the absence of NICD, RBPj/CBF-1 can also function in conjunction with transcriptional corepressor proteins to repress target gene expression. The most thoroughly characterized notch targets are the Hes and related Hey genes, which encode a family of basic helix–loop–helix (bHLH) transcriptional repressors [37]. Hes/Hey proteins then inhibit transcription of their target genes, such as *Ascl1*/*Mash1* and *Neurogenins*, thereby preventing undifferentiated precursor cells from achieving differentiated phenotypes. This core signal transduction pathway occurs in most notch-dependent processes and is known as the “canonical” pathway (Fig. 1) [1].

Notch signaling plays an essential role in maintenance of embryonic and adult NSCs. For example, it has been shown that a loss of function for notch receptors [38], Dll1 [39], Mind bomb [40], RBPj [11] or Hes transcriptional factors [41–43] resulted in the premature neuronal differentiation and rapid depletion of NSCs.

## Visualization of notch signaling to identify the signal-activated cells in tissues

To understand the precise physiological roles of notch signaling in the developing and adult nervous system, it is important to identify its target cells. When canonical notch signaling is activated, its activation levels are represented by the amount of NICD in nuclei. Thus, notch activation can be mapped, in situ, using a specific antibody that recognizes the processed form of NICD after it has been cleaved by Presenilins of the γ-secretase complex [44]. However, this procedure requires the cells to be fixed for immunohistochemical analysis and cannot, therefore, be used to detect notch activation in living tissues. Furthermore, the specific immunostaining for NICD requires an antigen-retrieval procedure that sometimes makes co-immunostaining for other marker proteins difficult. To overcome these limitations, several transgenic or knock-in mouse models were generated to monitor notch signaling activation and are used to identify notch signal-activated cell types in developing and adult nervous tissues.

In notch-activity reporter models, green fluorescent protein (GFP) or β-galactosidase protein (β-gal) encoded by the lacZ gene are used as read outs of signal activation. β-gal reporter detection requires tissues or cells to be fixed. Although X-gal staining to detect *LacZ* expression exhibits very high sensitivity and a low background signal, immunostaining for β-gal is occasionally difficult. As β-gal localizes in the cell body, it is difficult to clearly visualize cellular shapes, such as the radial processes of NSCs. GFP or its variants spread more diffusely throughout cells than β-gal, and fine cellular structures are more easily visualized by immunostaining for GFP. In addition to enhanced GFP (EGFP), enhanced yellow or cyan fluorescent proteins (EYFP or ECFP), are also employed in notch-activity reporter models; however, the emission spectra of these fluorescent proteins are relatively close, making their visual separation difficult with readily available imaging systems. Furthermore, due to the amino acid sequence similarity of GFP variants, it is impossible to separately identify each of the fluorescent reporter-expressing cells by immunohistochemistry using antibodies raised against GFP polypeptides. The red fluorescent protein from *Discosoma* sp. (DsRed) and its variants, however, are particularly useful for dual color imaging, and the anti-GFP and anti-DsRed antibodies do not cross-react with one another. In addition to DsRed, new reporter proteins with various fluorescence properties derived from other organisms have been identified and improved for application in cellular or developmental biology [45]. For example, in original Fucci cell cycle probes, new red- and green-emitting fluorescent proteins, mKO2 (the monomeric version of Kusabira Orange) and mAG (the monomeric version of Azami Green) are used [46]. Two DsRed variants, tdTomato and mCherry are widely used [47]. Fluorescent proteins with excitation and emission bands within the near-infrared optical window have been developed [48, 49], these near-infrared fluorescent proteins are useful especially in deep tissue visualization. The development of new transgenic or knock-in mouse models of notch signaling-activity with multicolor reporters will make possible more sophisticated imaging of notch signaling activity during various events in neurogenesis. Below, we review the widely used transgenic or knock-in reporter mouse models currently used in the field of notch signaling in neurogenesis.

### Transgenic notch reporter mouse

As mentioned above, notch receptors are transmembrane proteins activated by Delta and Jagged ligands. Upon activation, NICD is cleaved by γ-secretase and translocates into the nucleus to interact with the transcriptional regulator RBPj/CBF-1. In the developing nervous system, the NICD–RBPj complex activates target genes such as *Hes1* and *Hes5*, which antagonize proneural genes and neuronal differentiation.

**Fig. 3:**
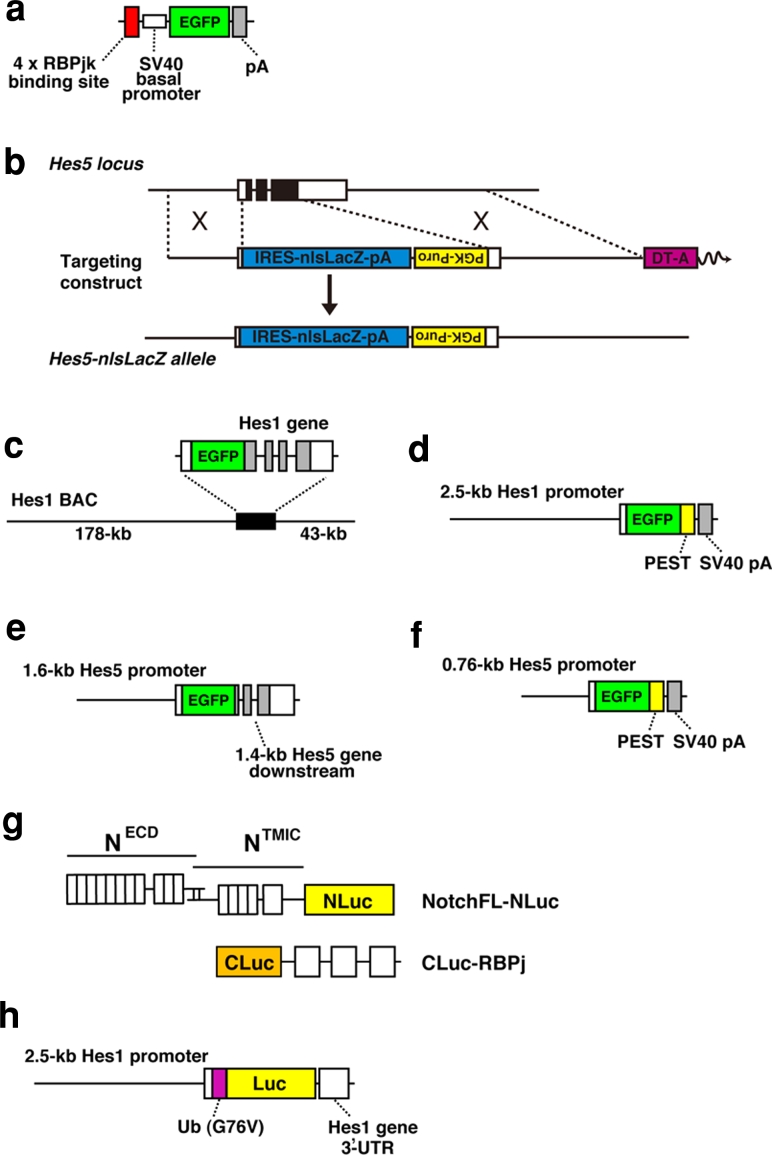
Transgenic constructs for monitoring the notch signaling activity. **a** TNR mouse. **b** Hes5-nlsLacZ knock-in mouse. **c** Hes1 BAC-EGFP mouse. **d** pHes1-d2EGFP mouse. **e** pHes5-EGFP mouse. **f** pHes5-d2EGFP mouse. **g** The LCI reporter for monitoring notch activation. **h** pHes1-Ub-Luc mouse. More detailed information is described in the text

The transgenic notch reporter (TNR) mouse was generated by the Gaiano lab (Fig. 3a) [50]. In the transgenic construct, an EGFP sequence was placed under the control of four tandem copies of the RBPj binding consensus sequence upstream of the SV40 basal promoter. The TNR transgene faithfully reports RBPj activity. EGFP expression can be upregulated in transgenic mouse embryonic fibroblasts after infection with activated NOTCH1–3, or an activated form of RBPj (RBPj-VP16). In addition, EGFP expression can be inhibited by shRNA-mediated knockdown of *RBPj*, or by treatment with a γ-secretase inhibitor. RBPj activity-dependent EGFP expression was also confirmed in NSCs in vivo [50]. Therefore, in the TNR transgenic mouse, EGFP expression is specifically induced in cells with pathway activation, and thus this transgenic mouse strain is very useful for characterizing endogenous notch signaling.

In the TNR transgenic mouse strain, EGFP expression is present in a wide variety of cell/tissue types during development and in NSCs in the adult. In the developing nervous system, EGFP is expressed in the germinal zone of the brain and spinal cord in a pattern similar to the neural progenitor markers Nestin and CD133 [50]. Surprisingly, in the telencephalic VZ of the TNR mouse embryos, EGFP expression was not uniform, indicating heterogeneous activation of notch signaling. Gaiano’s work using TNR mice demonstrated that signal transduction is differentially regulated in these specific subsets of progenitor cells. Detailed analysis showed that NSCs and INPs are able to respond to notch receptor activation, but the canonical notch signaling via RBPj and subsequent *Hes* activation is selectively induced only in NSCs, whereas INPs exhibit attenuated RBPj signaling. Analyses using both transgenic and transient in vivo reporter assays revealed that NSCs and INPs coexist in the telencephalic VZ and that they can be prospectively separated on the basis of RBPj activity [4, 50]. However, little is currently known about how differential activation of notch signaling is achieved among these specific subsets of progenitor cells.

In the TNR mouse strain, the location of EGFP expression is consistent with notch signaling pathway elements/genes and appears to faithfully reflect canonical (RBPj-mediated) notch activity. Similar to TNR mice, Souilhol et al. [51] reported NAS transgenic mouse line carrying a construct consisting of nlsLacZ gene under the control of multiple RBPj binding consensus sequence and a minimal promoter. In addition to neurogenesis research, these mice have also been used in hematopoietic stem cell research, and in studies examining angiogenesis and hair follicle formation [52–54]. These works indicate that the differential activation of notch signaling, in particular with respect to RBPj activation, may be a mechanism used to distinguish between stem and progenitor cell subtypes in many tissues.

### Hes5-nlsLacZ knock-in mouse

The most thoroughly characterized notch targets are the Hes and related Hey genes, which encode a family of basic helix–loop–helix (bHLH) transcriptional repressors [37]. Among them, *Hes5* expression is the most highly dependent on notch signaling. Other notch target genes, including *Hes1*, *Hes3*, and *Hes7*, are also regulated by signaling pathways other than notch signaling [55–57]; therefore, *Hes5* is a more faithful notch effector.

To monitor notch signaling activity using *Hes5* expression, our group visualized the expression of *Hes5* by knocking in the nlsLacZ cDNA at the *Hes5* locus (Hes5-nlsLacZ mice) (Fig. 3b) [11]. In this mouse strain, nuclear-localized β-gal reporter protein encoded by the lacZ gene is expressed from the endogenous *Hes5* promoter and mimics endogenous *Hes5* expression. The resultant β-gal was highly expressed in the VZ of the developing central nervous system, as expected. β-gal was also expressed in the two germinal zones of the adult brain: the SVZ of the lateral ventricles and the SGZ of the hippocampal dentate gyrus [11].

Notch signaling is highly activated in type B cells of the SVZ/lateral ventricle and type 1 cells of the SGZ/dentate gyrus. In the SVZ of Hes5-nlsLacZ mice, almost all β-gal-positive cells expressed GFAP, a type B cell and astrocyte marker. The majority of Mash1-positive type C cells were negative for β-gal expression, while a small number of co-expressing cells were observed. In addition, after prolonged exposure to BrdU, only a few β-gal-positive cells were labeled with BrdU, suggesting that the majority of *Hes5*-expressing cells are not actively dividing. However, none of the ependymal cells (S100b+) or neuroblasts (DCX+) expressed β-gal in the Hes5-nlsLacZ mice. Thus, *LacZ* expression occurred in almost all type B cells as well as in astrocytes and a small number of type C cells, but not in others. Because the β-gal protein is stable, it is likely that the expression remained transiently in type C cells even after the *Hes5* promoter was repressed, suggesting that notch signaling is active mostly in type B cells [11]. Analysis of the hippocampal dentate gyrus from the TNR mice revealed that notch signaling is also highly activated in NSCs of this neurogenic region [13]. Under normal conditions, notch-activated adult NSCs are largely quiescent, and only restricted subsets seem to be proliferating and generating adult born neurons through transit-amplifying cells. Through the conditional inactivation of *RBPj*, it was shown that notch signaling is absolutely required for the maintenance of NSCs and the proper control of neurogenesis in both embryonic and adult brains [11–15]. Although *Hes5* is also expressed in non-stem cell astrocytes throughout the adult brain as well as in NSCs, the Hes5 reporter mouse strain is an important candidate tool to prospectively identify and separate NSCs in the adult brain.

Notch signaling in the nervous system has been most studied in the context of cell fate specification during development. Recently, it was reported that, in the adult mouse brain, notch signaling is activated in neurons by synaptic activity [58]. NOTCH1 and its ligand Jag1 are present at the synapse, and expressions of both are increased in response to neuronal activity. Furthermore, neuronal notch signaling is positively regulated by Arc/Arg3.1, an activity-induced protein required for synaptic plasticity, and NOTCH1 is required for the synaptic plasticity that contributes to memory formation. Obvious expression of Hes genes in post-mitotic neurons has not been reported [but see 59]. Thus, neuronal notch signaling might contribute to synaptic plasticity through non-canonical, downstream target genes, although it is possible that Hes/Hey genes are expressed in neurons at very low-levels. In the TNR mice, notch signaling activity is visualized as the transcriptional activity of the NICD–RBPj–Maml complex. The TNR reporter is not susceptible to Hes negative autoregulation because the TNR promoter does not contain Hes-binding sites unlike the *Hes* promoters (see below), and therefore activation of TNR reporter expression could be detectable in neurons even though the endogenous Hes expression is undetectable. Thus, the combined use of TNR and Hes5 reporter mice will provide the important information about the employment of the downstream target genes by activation of notch signaling.

### Hes1 BAC-EGFP mouse/pHes1-d2EGFP mouse

The Hes1 BAC-EGFP transgenic mouse was generated with a bacterial artificial chromosome (BAC), in which the coding sequence of EGFP was inserted into the 5′-UTR of the Hes1 gene (Fig. 3c) [60]. Immunofluorescent staining for Hes1 and EGFP on adjacent sections confirmed co-expression of EGFP with Hes1 in progenitor cells of the developing nervous system. As EGFP expression in this transgenic strain is regulated by a huge regulatory sequence, 224 kb of chromosome 16 including the Hes1 gene, the EGFP expression is supposed to reliably reflect *Hes1* expression sites. However, EGFP has a much longer half-life than the tightly regulated Hes1 transcription factor. Compared with the half-life of *Hes1* mRNA and protein (~20 min), normal EGFP is so stable that significant EGFP might remain after the *Hes1* promoter is downregulated.

In the pHes1-d2EGFP transgenic mouse (Fig. 3d) [61, 62], destabilized EGFP (d2EGFP), which has a half-life of ~2 h due to an attached PEST sequence, is driven by the 2.5-kb *Hes1* promoter. d2EGFP is suitable for the precise fluorescent imaging of *Hes1* promoter-activated cells. In the developing brains of these mice, d2EGFP expression was restricted to undifferentiated progenitor cells in the VZ, including radial glial cells, and FACS-sorted d2EGFP-positive cells efficiently generated neurospheres. d2EGFP expression in the telencephalic VZ persisted until early postnatal life, and some d2EGFP-positive cells were still present in the SVZ of the lateral ventricle and the hippocampal dentate gyrus in the adult brain. Although, d2EGFP expression in the pHes1-d2EGFP mice largely mimics the pattern of endogenous *Hes1* mRNA expression in the developing nervous system, there are small discrepancies [61]. Furthermore, *Hes1* expression is also regulated by signaling pathways other than notch signaling, such as sonic hedgehog or JAK-STAT signaling [55, 63]. Hence, *Hes5* knock-in or *Hes5* promoter transgenic mice are more faithful reporters of the contextual canonical notch signaling, although *Hes5* expression is also modified by other signaling pathways, such as BMP signaling [64]. Both Hes1 BAC-EGFP and pHes1-d2EGFP transgenic mice are still very useful tools to mark, isolate, and enrich NSCs based on their EGFP fluorescence.

### pHes5-EGFP mouse/pHes5-d2EGFP mouse

To identify cells with active notch signaling in the developing nervous system, Verdon Taylor’s group generated a notch reporter transgenic mouse using the *Hes5* promoter and regulatory elements to drive expression of EGFP (Fig. 3e) [65]. The transgenic construct (pHes5-EGFP) contains 1.6 kb of the 5′-flanking sequence of the mouse Hes5 gene, including the putative promoter and regulatory elements, and 1.4 kb of downstream sequence that includes the three exons and two introns of the Hes5 gene [65]. Four putative RBPj binding sites in the 1.6-kb promoter and two in exon III of *Hes5* were identified. The EGFP coding sequence, including a translational stop codon, was inserted downstream of the translation initiation site of *Hes5*. Therefore, the transgene generates a transcript comprised of the 5′-untranslated region (UTR) of *Hes5*, the EGFP coding region, and the remaining *Hes5* transcript, including the endogenous 3′-UTR and polyadenylation signal. It is possible that *Hes5* mRNA stability is regulated at the post-transcriptional level, for example by micro-RNA; therefore, it is expected that inclusion of the endogenous *Hes5* transcript and particularly the 3′-UTR resulted in the rapid downregulation of Hes5-EGFP expression as cells leave the VZ. Similarly to Hes1 BAC-EGFP and pHes1-d2EGFP transgenic mice, pHes5-EGFP expression is restricted to germinal zones of the developing brain. Marker protein co-expression and cell sorting confirmed that EGFP is expressed by progenitors but not by immature neurons. Furthermore, purified EGFP-positive cells from the neural tubes exhibited self-renewal capability and multipotency. pHes5-EGFP expression was highly dependent on notch signaling, as EGFP expression was completely lost in NOTCH1-deficient embryos [65]. Although, EGFP expression in the transgenic mice largely mimics the pattern of endogenous Hes5 expression, additional reporter models using BAC or knock-in approaches might be needed to address the exact monitoring Hes5 expression. Indeed, Hes5 BAC-EGFP transgenic mice were generated and characterized by GENSAT project [66]. We also generated pHes5-d2EGFP mice (Fig. 3f) [61], in which d2EGFP expression is expressed under the putative *Hes5* promoter. However, in this transgenic construct a 0.76-kb short 5′-upstream region of *Hes5* was employed, which resulted in some degree of ectopic expression and did not completely reflect the expression of the endogenous Hes5 gene, although d2EGFP expression was restricted to subsets of NSCs.

Using pHes5-EGFP transgenic mice, Taylor and Giachino’s group showed that NSCs in the adult dentate gyrus depend on canonical notch/RBPj signaling and express *Hes5* whereas intermediate progenitors and neuronal precursors do not [12]. Visualization of canonical notch/RBPj signaling and *Hes5* expression labeled a fraction of the Sox2-positive progenitors in the dentate gyrus, and based on their in vivo and in vitro characteristics, these pHes5-EGFP-positive cells were defined as NSCs. However, it is still not clear whether all pHes5-EGFP-positive cells in the SGZ are NSCs. In the dentate gyrus of the hippocampus, NSCs represent two morphologically distinct (radial and horizontal) populations that differ in proliferation levels. Radial NSCs are believed to divide asymmetrically to give rise to more committed daughter cell types through the transition to horizontal NSCs [32]. However, the precise lineage relationships between radial and horizontal NSCs are still controversial [67]. Lugert et al. [12] found that pHes5-EGFP labeled both radial and horizontal NSCs in the SGZ. Using pHes5-EGFP expression, they visualized radial and horizontal NSCs and characterized strikingly different proliferative behaviors of these cells after neurogenic stimuli, such as voluntary exercises and seizures, or aging. Running was found to stimulate proliferation of radial precursors, whereas seizures, induced by intraperitoneal kainic acid injections, activated both populations and expanded the horizontal population. Surprisingly, the number of non-dividing NSCs expressing pHes5-EGFP (both radial and horizontal) was not significantly changed with aging, and the induced seizures in aged mice activated the proliferation of pHes5-EGFP-expressing cells [32].

In addition to the above reporter mice, a number of groups have generated reporter plasmids or viral vectors using 5′-flanking regions of the Hes genes and their homologs to follow notch signaling in various vertebrates [68–70]. Kohyama et al. [68] applied Venus (and its destabilized form dVenus) instead of EGFP. Venus is an EYFP variant that exhibits fast and efficient maturation, and a strong fluorescence intensity [45]. One of the limitations of the reporter constructs and mouse lines discussed above is the perdurance of the reporter proteins. Depending on the mRNA and protein half-lives of reporter constructs, the reporter might not exhibit correct temporal expression. One way to avoid this problem is to use constructs with mRNA degradation-promoting (i.e., ARE) or peptide degradation-domain attached reporter (e.g., PEST) sequences [61, 71]. Another strategy is to design the knock-in allele to synthesize a fusion protein between an endogenous protein product and a fast-maturing fluorescent protein. In the latter strategy, by visualizing the live fluorescence of fusion proteins, we might be able to precisely monitor the spatiotemporal dynamics of important component molecules of notch signaling. Although these strategies have not been commonly applied to mice, possibly due to the trade off of reduced reporter sensitivity, it is necessary to visualize notch signal activity in real-time, to thoroughly understand when, where, and how notch signaling is dynamically regulated [4].

## Real-time imaging of notch activation with a luciferase complementation method

Recently, Ilagan et al. [72] reported real-time imaging of notch signal activation using luciferase complementation imaging method (LCI), which was originally developed by Luker et al. [73]. In this technique, N- and C-terminal fragments of *Firefly* luciferase (NLuc and CLuc), which have no activity on their own, are reassembled into a functional enzyme by the interaction of proteins to which they are fused. Ilagan et al. applied this technique to real-time visualization of NICD and RBPj interaction, which occurs upon activation of notch signaling.

NLuc and CLuc were fused to NOTCH1 and RBPj, respectively (Fig. 3g), and the formation and turnover of the NICD–RBPj complex were monitored by the luciferase activity in the real-time assay system. Upon activation of notch signaling, the luciferase activity was detectable in nuclei of living cells. This method is useful for measurement of the pharmacodynamics of various different inhibitors and high-throughput screening for notch activators. However, this method may require further improvements to detect activation of other notch receptors (Noch2, 3 and 4) and to obtain higher sensitivity and temporal-spatial resolution for real-time imaging at the single cell level.

## Real-time imaging of the spatiotemporal dynamics of notch signaling activation state using super-unstable luciferase reporters

**Fig. 4:**
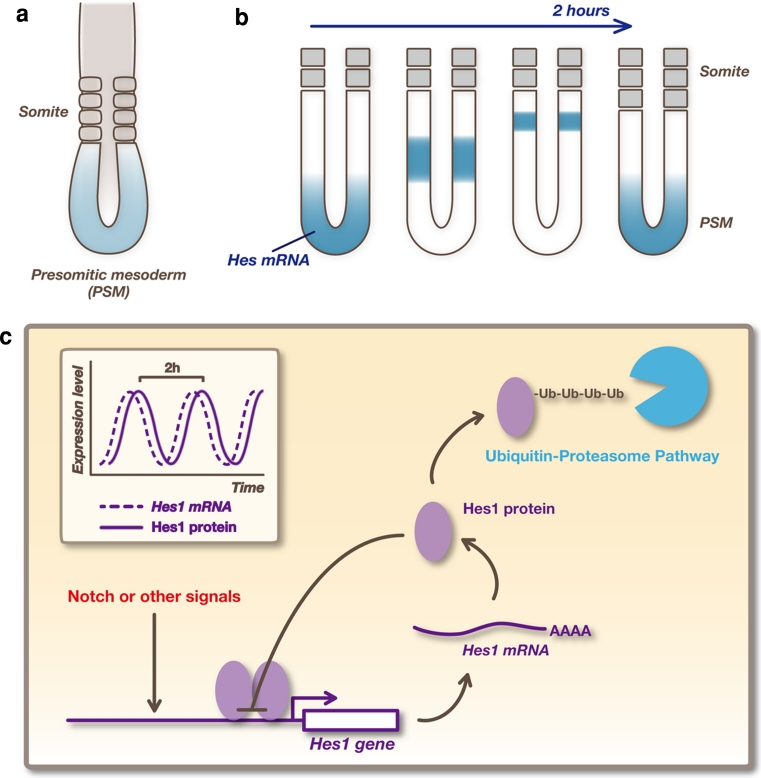
Oscillatory expression of Hes genes. **a** Somites form periodically by segmentation of the anterior region of the presomitic mesoderm (PSM). **b** *Hes* expression is periodically propagated, like a wave, from the posterior end to the anterior region of the PSM, and each wave leads to the generation of a pair of somites. **c** Oscillatory expression of *Hes1* is regulated by negative feedback. Promoter activation induces the production of Hes1 protein, which represses expression of its own gene. Then, both *Hes1* mRNA and Hes1 protein disappear rapidly because they have very short half-lives, allowing the next round of expression. In this way, *Hes1* expression autonomously oscillates

Various notch signaling molecules display an ultradian oscillatory expression in various cell types, such as fibroblasts, NSCs, and embryonic stem cells [3]. Oscillation of notch signaling has been most extensively analyzed in the presomitic mesoderm (PSM) [74, 75]. Somites, the segmental units that later give rise to the vertebrae, ribs, skeletal muscles, and dermis, are formed by the segmentation of the anterior region of the PSM (Fig. 4a). This event is repeated every 2 h in mouse embryos. It has been suggested that this periodic event is controlled by a biological clock, called the segmentation clock. It was reported that the expressions of *Hes1*, *Hes5*, and *Hes7* are periodically propagated in a wave-like fashion initiating at the posterior end and moving towards the anterior region of the PSM (Fig. 4b). Each wave leads to the generation of a pair of somites. In addition, the expression of the glycosyltransferase lunatic fringe (*Lfng*), which regulates notch activity, also oscillates in phase with *Hes* expression. *Lfng* periodically inhibits notch signaling and thereby generates oscillations in notch activity, which may in turn influence *Hes* oscillation.

In many cultured cells, including fibroblasts, myoblasts, and neuroblasts, a synchronized oscillatory *Hes1* expression can be induced following serum stimulation or notch activation [63, 76, 77]. *Hes1* oscillation is cell-autonomous and depends on negative autoregulation (Fig. 4c). After induction, Hes1 protein represses the expression of its own gene by directly binding to its promoter. This repression is short-lived due to the short half-life of the mRNA and protein. In this way, *Hes1* autonomously initiates oscillatory expression with a periodicity of approximately 2 h, suggesting that *Hes1* acts as a biological clock.

We have established a real-time, bioluminescence imaging system of *Hes* expression using super-unstable luciferase reporters (Fig. 3h) [78–80]. Because the half-life of Hes1 protein is ~20 min, that of the reporter should be 20 min or less [76]. Otherwise, the reporter protein will accumulate after several cycles of oscillation. To overcome these problems, we used the ubiquitinated *Firefly* luciferase (Ub-Luc) as a reporter, which reacts to such rapid synthesis and degradation processes [81]. This luciferase was fused at its N-terminus to one copy of a mutant ubiquitin (G76V) that resists cleavage by ubiquitin hydrolases. The resultant Ub-Luc is extremely unstable with a half-life of less than 10 min [78]. Compared with fluorescent proteins, such as EGFP or DsRed, the maturation time of luciferase is more rapid. We inserted the Ub-Luc coding sequence in the transgenic construct that contained 2.5 kb of the 5′ flanking sequence of the mouse Hes1 gene, which contains sites for both notch induction and negative feedback, and 0.5 kb of downstream sequence that includes the 3′-UTR region of the Hes1 gene. Real-time imaging of the bioluminescence revealed that *Hes1* promoter-driven Ub-Luc reporter expression oscillated in individual cultured fibroblasts and PSM cells [78].

Although a role for oscillatory *Hes1* expression has been extensively analyzed in somitogenesis, only recently has such an oscillatory pattern been observed in the embryonic nervous system [6, 80]. Real-time imaging and quantitative measurement of the bioluminescence signal of pHes1-Ub-Luc revealed the oscillation cycle of *Hes1* in telencephalic NSCs to be ~2 h, consistent with that observed in other settings [80]. Notably, Ngn2 and Dll1 both display inverse correlations with Hes1 expression levels in NSCs, suggesting that Ngn2 and Dll1 expression also oscillates in these cells but with opposite phases to Hes1 oscillation. Real-time imaging showed that *Ngn2* and *Dll1* are indeed expressed in an oscillatory manner by dividing NSCs. It is likely that Ngn2 oscillation is regulated by Hes1 oscillation, while Dll1 oscillation is under the control of cyclic induction by Ngn2 and cyclic repression by Hes1. Dll1 expression then leads to activation of notch signaling between adjacent NSCs. Thus, *Hes1*-driven *Ngn2* and *Dll1* oscillations are essential for maintenance of a group of cells in an undifferentiated state by mutual activation of notch signaling [6, 80]. Therefore, real-time bioluminescence imaging of notch signaling activity revealed that it is under the control of more complex, and dynamic regulation than previously thought. Further study is required to fully understand the importance of oscillating notch activity and *Hes* expression in NSCs, particularly what kinds of biological events are dynamically coupled to *Hes* oscillation, for example, cell-fate determination, cell cycle progression, and regulation of adult NSC quiescence [4]. To understand these events, visualization of *Hes1* promoter activity as well as real-time imaging of Hes protein dynamics are definitely required.

As discussed above, luciferases are the most suitable reporters for the quantitative measurement of gene expression because their sensitivity and range of linear response are superior to those of other typical reporters, including β-gal, chloramphenicol acetyltransferase (CAT), β-glucuronidase, and fluorescent proteins [82, 83]. Another advantage of luciferases over fluorescent reporters is that they do not require exogenous illumination because they catalyze luciferins to emit light directly, so the background emission from samples is extremely low.

However, the current limitation of real-time imaging of luciferases is their reduced color variations compared with the large repertoire of fluorescent proteins, making it difficult to simultaneously monitor more than one gene. An in vitro, tricolor-reporter assay system was developed, in which expression of three genes can be monitored simultaneously by splitting the emissions from green-, orange-, and red-emitting beetle luciferases with optical filters [84]. A dual-color real-time monitoring of the expression of two genes was also developed that uses the green-emitting luciferase derived from *Rhagophthalmus ohbai* (λmax = 550 nm) and the red-emitting luciferase derived from *Phrixothrix hirtus* (λmax = 630 nm) [85]. These green- and red-emitting luciferases use the common substrate, d-luciferin, and their emissions are then separated by optical filters. However, the bioluminescence spectra of these two luciferases overlap, and the complete separation with optical filters leads to the significant loss of each bioluminescence. An alternative dual-reporter assay involving two luciferases (*Cypridina* luciferase and *Gaussia* luciferase) was also developed [86]. However, these two luciferases are secreted outside the cell and are not suitable for real-time observation by the microscopy cameras. Further improvement in these luciferase variants will make multi-color bioluminescence imaging a more powerful strategy in real-time imaging of notch signaling during various cellular and developmental events.

Another drawback of luciferase-based imaging is that bioluminescence signals are too dim to be measured in real-time; therefore, bioluminescence imaging generally requires longer exposure than fluorescence imaging that takes less than one second. Especially, destabilized luciferases, such as Ub-Luc, require extremely long exposure times of more than several minutes [78]. To overcome these drawbacks, efficient bioluminescence resonance energy transfer (BRET) from a bioluminescent protein to a fluorescent protein with high fluorescent quantum yield has been utilized to enhance luminescence intensity. For example, an autoilluminating fluorescent protein, eBAF-Y, has been developed that is based on the highly efficient BRET between *Renilla* luciferase and EYFP, and emits a brighter signal than the *Renilla* luciferase [87]. The availability of more efficient autoilluminating fluorescent proteins will extend current capabilities and may allow single-cell imaging in near real-time without external light illumination.

A wide range of notch ligands can bind and activate the notch receptors. The ligand-induced activation of notch signaling is dynamically regulated by endocytic trafficking, which can be modulated by the different ubiquitin ligases [34]. However, ligand–receptor interactions and their regulatory mechanisms in neural tissues are largely unknown. It is still not completely clear where the hotspot of ligand–receptor interactions is in highly polarized NSCs of the developing and adult brains. It is expected that further improvement of technologies to analyze ligand–receptor complexes in intact cells and living animals will advance research into the dynamic regulation of ligand–receptor interactions of notch signaling.

## Conclusions

It is now clear that the notch signaling pathway is under more complex and dynamic regulation than previously thought. How these multiple regulatory mechanisms are coordinated in the developing nervous system remains to be determined. Advances in genetic visualization of notch signaling will greatly contribute to further understanding how notch signaling is regulated during neurogenesis in the developing brain. As notch signaling is essential for NSC maintenance in the adult brain and for prolonged neurogenesis throughout life, these observations indicate that dysfunction of notch signaling might contribute to stem cell loss and reduced neurogenesis during aging. The further analysis of notch signaling regulation will add to our understanding of the fundamental mechanisms of adult neurogenesis, which may lead to the development of novel therapies for functional recovery after disease, trauma, or pathological aging.
